# AI‐assisted automation will reduce the need for medical physics trainees and entry‐level workforce

**DOI:** 10.1002/acm2.70756

**Published:** 2026-08-20

**Authors:** Junyi Xia, Alan McMillan, Dongxu Wang

**Affiliations:** ^1^ Department of Radiation Oncology Icahn School of Medicine at Mount Sinai New York New York USA; ^2^ Department of Radiology and Department of Medical Physics University of Wisconsin‐Madison Madison Wisconsin USA; ^3^ Department of Medical Physics Memorial Sloan Kettering Cancer Center New York New York USA

**Keywords:** artificial intelligence, medical physicist, trainee, workforce

## 1 INTRODUCTION

Artificial intelligence (AI) is reshaping many professions, and medical physics is no exception. Deep‐learning auto‐segmentation, automated treatment planning, AI‐assisted chart review, and emerging agentic systems can now perform, or meaningfully accelerate, many tasks that have traditionally filled the days of physicists. This raises a workforce question for our profession: if each physicist can accomplish substantially more with AI support, will we still need as many entry‐level physicists and trainees as we do today? The trainee and entry‐level workforce span two connected labor markets: graduate students and postdoctoral researchers in academia, and residents and junior physicists in the clinic. AI touches both — automating research tasks like data curation and coding on one side, and clinical tasks like contouring, planning, and chart review on the other.

In this Parallel/Opposed Editorial, two medical physicists take opposing positions on the proposition “AI‐assisted automation will reduce the need for medical physics trainees and entry‐level workforce”. Dr. Junyi Xia argues for the proposition, contending that rising per‐physicist productivity will gradually contract hiring at the margin. Dr. Alan McMillan argues against it, contending that AI is a cognitive multiplier that will expand the scope, complexity, and demand for trained experts. Each presents an opening statement, followed by a rebuttal.

## 2 OPENING STATEMENTS

### 2.1 Dr. Junyi Xia, for the proposition

AI is already changing how work is done across many industries. AI systems can now write software code, support decision‐making, summarize documents, and analyze images. Emerging agentic AI systems can work as a team to carry out tasks from start to finish. As more capable AI systems move closer to general‐purpose use, medical physicists, like many other professions, will face a practical question: if each of us can do substantially more with AI support, how many entry‐level physicists and trainees will our field actually need?

This general shift is already visible in software engineering. In 2025, roughly 20%–30% of code in Microsoft repositories was being written by AI tools.^1^ Google's numbers appear even more striking, with reports stating that 75% of Google's new code is now AI‐generated.^2^ Using AI, one engineer can now generate, review, and maintain work that might previously have required a larger team. At the same time, many major technology companies are reducing or restructuring their workforces. Meta recently announced plans to cut about 8000 jobs, roughly 10% of its workforce, while also leaving thousands of roles unfilled.^3^ Microsoft has offered voluntary buyouts to about 8750 U.S. employees, approximately 7% of its U.S. workforce.^3^ These reductions have multiple causes, but the overall signal is hard to miss: when AI enables each professional to do more, organizations begin to ask whether the same work can be done with fewer people.

Our field may seem very different from software engineering. However, in many ways, radiation oncology has similar characteristics: it is technology‐heavy, data‐rich, protocol‐driven, and deeply dependent on complex software and hardware systems. Many tasks performed by trainees and junior physicists are routine, labor‐intensive, and essential to clinical operations: treatment planning support, quality assurance, documentation, cross‐checking, and chart review. These are exactly the kinds of tasks that AI systems are increasingly able to support. AI‐assisted automation improves efficiency by taking over portions of the routine work that currently consumes much of a physicist's day. Auto‐contouring software can reduce the time spent on manual contouring. AI‐assisted planning can improve planning efficiency while achieving high plan quality. Automated chart‐checking tools can flag inconsistencies in treatment plans and treatment records. Machine‐learning systems can monitor linac performance and identify trends before they become obvious in routine reviews. Even routine documentation can increasingly be drafted by AI. Each of these tools may save only part of a physicist's time. Together, however, the effect could be substantial. A physicist who once spent hours reviewing charts and plans may instead review dashboards, trends, and outlier alerts. A physicist who once performed multiple iterations of manual treatment planning may instead evaluate AI‐generated plans and intervene only when a case is complex or when the AI‐generated plan does not meet clinical criteria. The physicist remains responsible, but the amount of work one physicist can oversee increases significantly.

The next wave of agentic AI makes this even more consequential. Agentic AI systems will not simply complete individual tasks; they will coordinate many tasks across the clinical workflow. Such a system could receive a prescription, evaluate contours, generate treatment plans, verify treatment parameters, analyze patient‐specific QA results, draft documentation, and escalate only unusual or high‐risk situations to a physicist. This would not remove the physicist's responsibility, but it would greatly expand the physicist's supervisory capacity. This is an important difference from earlier technological advances in radiation oncology. In the past, new technology often increased physics workload. IMRT, IGRT, MRI‐Linac, and proton therapy all brought new procedures, workflows, commissioning needs, and QA requirements. More advanced technology often meant more work for physicists. AI‐assisted automation is different. Its purpose is to absorb routine work, manage complexity, and increase the productivity of the people supervising the system.

As agentic AI and more general AI capabilities mature, AI‐assisted automation will increase the efficiency of individual physicists. Even a modest increase in productivity can meaningfully affect workforce demand. In practice, departments make hiring decisions at the margin. If existing staff can absorb incremental workload through improved efficiency, the need to recruit additional physicists or expand residency programs may be reduced or delayed. Over time, these small adjustments accumulate, leading to a gradual contraction in the training pipeline. The issue is therefore not sudden workforce displacement, but a steady reduction in demand for new entrants. These trends suggest that AI‐assisted automation is likely to reduce the demand for medical physics trainees and junior physicists in the long term.

### 2.2 Dr. Alan McMillan, against the proposition

The development of AI is a profound landmark of human achievement. As of the writing of this statement in 2026, AI has the ability to meet or exceed human performance across many domains^4^; this gives us reason to ponder how it will impact our field, and the future of our medical physics trainees. However, will the availability of technologies for deep learning‐based automatic segmentation, treatment planning, and dynamic adaption lead to a reduction in trainees and entry‐level positions? Furthermore, will the availability to perform AI‐enabled programming (“vibe coding”) and data curation limit the need for new human intelligence in these fields? I choose to have an optimistic view of the impact of AI on our need for more trainees and entry‐level workforce. AI is a cognitive multiplier, giving unparalleled skills and potential for synthesis and expression and these capabilities will move the field towards unprecedented capability and complexity. This will expand, not constrain, the demand for highly trained graduates.

One notable example from history of how technical innovation that increases the efficiency of a resource, can actually increase overall total utilization is Jevon's Paradox.^5^ In the mid 1800s the development of novel steam engine technology greatly increased efficiency and thus reduced the amount of coal needed. However, instead of decreasing the demand for coal, overall utilization actually increased. While this is often cited in the context of economic theory of supply and demand, perhaps it will be applicable to the human capital in our field as well. Automation historically complements human labor, inducing demand for workers to perform higher‐order, non‐automatable tasks.^6^

In medical physics research, we are not limited by the number of testable hypotheses we can develop. Instead, we are limited by time and resources. Historically, students may spend months manually contouring organs and tumor volumes to painstakingly curate a database by hand. Now AI foundation models can automatically segment thousands of scans, harmonize data, and generate code to analyze the results in vastly shorter time frames.^7^, ^8^, ^9^ Thus if a retrospective analysis with 50 or 100 patients can readily be automated, this moves the baseline for impactful publications to thousands or more patients, integrating data across multiple domains, and institutions.^10^ So instead of replacing the status quo with automation, more automation will drive the capability and quality expectations even higher.

Automated organ segmentations for radiation therapy planning have been clinically available since approximately 2010 and with the development of deep learning this has greatly expanded within the last 10 years.^11^, ^12^, ^13^ As of today, this has not created a situation in which medical physicists are being replaced. In fact, we see an increase in vastly more complicated therapy techniques such as stereotactic body radiotherapy (SBRT), adaptive radiotherapy (ART), FLASH radiotherapy, and the use of MR‐Linacs to dynamically track and manage motion during treatment. The report from the American Association of Physicists in Medicine (AAPM) Task Group 275^14^ on physics plans and chart review notes that automation will allow physicists to “have more time to devote to other aspects of the chart check that do not lend themselves to automation such as overall plan quality, review of prior treatment, laterality confirmation, or issues related to image guidance.” Rather than replacing humans in this role, medical physicists will find new ways to advance our capabilities. Therefore, it is hard to justify that we will need fewer trained experts in this field, when we still have significant disease and mortality.

There remain legitimate concerns about the use of AI leading to automation bias,^15^ where the use of advanced technology implores users to willingly accept the outputs without meaningful oversight, as well as de‐skilling,^16^ where the workforce may lose fundamental knowledge because the tasks that require it can be fully automated. However, these concerns are only major if the field stagnates and there is no progress. Unfortunately we have not yet come close to curing cancer,^17^ despite increasing survival for many types of cancer. Since progress in science relies on incremental developments upon foundational work (*nani gigantum humeris insidentes*), it seems highly unlikely that academia will forget the fundamentals in pursuit of the latest cures and technological advances.

Therefore, the assertion that AI‐assisted automation will precipitate a long‐term reduction in medical physics trainees and entry‐level workforce mischaracterizes the engine of academia; and academia is where medical physicists start their career in this profession, which requires at minimum a Master's degree and has close to 50% of the training residents holding a doctoral degree.^18^ Principal investigators secure resources to generate novel intellectual property, solve complex physical problems, and drive clinical translation. They are not hiring only script‐writers or manual image‐segmenters. By stripping away the friction of data curation, coding, and modeling, broad AI tools will trigger a massive expansion in the scale and impact of research and development. Future graduate students and postdoctoral trainees will not be displaced. Instead, they will evolve into sophisticated scientific architects, uniquely equipped to validate and direct arrays of AI tools to solve previously intractable healthcare challenges. The trainee workforce will not decrease to meet the demands of this new frontier.

## 3 REBUTTALS

### 3.1 Dr. Junyi Xia, for the proposition

I enjoyed Dr. McMillan's opening statement, and we agree on more than we disagree on. AI is a novel cognitive multiplier, and I share his optimism about where the field is going. The disagreement is narrower than it looks. He thinks multiplying each physicist's output will increase the number of physicists we hire. I'm not convinced his assumption will get us there.

Take Jevons' paradox.^5^ Coal was a commodity with wide downstream uses, so cheaper steam power created entirely new demand. Clinical medical physics doesn't work that way. Our demand comes from the number of patients treated, staffing regulations, and reimbursement. Faster auto‐contouring does not produce more cancer patients. And it's worth remembering what actually rebounded in Jevons' case: it was coal, not coal miners. As output per worker climbed, mining employment fell. More physics output does not have to mean more physicists.

Automation has historically complemented labor by moving people toward more intelligent work that machines couldn't do. That held because the cognitive work stayed with humans. Modern agentic AI systems will perform the intelligent work humans used to do: planning, reasoning, and decision‐making. Once that cognitive work can be automated, the old reassurance gets shakier.

Dr. McMillan is right that auto‐segmentation has been around since 2010 and we weren't replaced, and SBRT, ART, FLASH, and MR‐Linac entered clinical practice over the same period. But every one of those added manual work. New technology in our field has almost always meant more for physicists to do. Agentic AI is the first broadly applicable technology that could automate every part of our work, from contouring, planning, verification, and QA to documentation, needing our attention only on the exceptions. TG‐275 itself frames automation as freeing physicist time. At the margin, that freed time gets absorbed by current staff long before it justifies hiring someone new.

I also want to point out that much of Dr. McMillan's case is really about trainee's work in academia. Dr. McMillan is right that academia is where every physicist's career begins: all of us pass through graduate school, and nearly half of residents hold a doctoral degree.^1^ ^8^ But that is precisely why the research and clinical labor markets are not parallel tracks—they are a single pipeline, and its width is ultimately set at the clinical end. AI may well expand what each graduate student can attempt, as Dr. McMillan describes. Yet eventually, the number of trainee positions our profession sustains depends on where trainees ultimately land, and most land in clinical roles governed by patient volume, regulation, and reimbursement rather than by the supply of testable hypotheses. Today's tight job market does not provide reassurance either. In the past few years, the demand for physicists has been known to exceed supply, which means departments with unfilled positions have every incentive to adopt automation just to cope. When AI lets existing staff absorb that workload, the vacancy is not filled later—it quietly ceases to exist. In a shortage, efficiency gains do not cause layoffs; they dissolve open positions before anyone counts them. The shortage will mask the contraction until the contraction is well underway.

I'm not predicting that anyone loses a job tomorrow, and I don't think Dr. McMillan and I are far apart on what we value. My point is that departments hire at the margin. As efficiency lets current staff take on more tasks, the next hire gets postponed and the pipeline grows more slowly. Small adjustments add up, and that is what I expect over time.

### 3.2 Dr. Alan McMillan, against the proposition

The proposition that AI will reduce the need for medical physics trainees and entry‐level workforce assumes a static ceiling of clinical capability: if AI completes today's tasks faster, clinics will need fewer physicists. When agentic AI and deep‐learning auto‐segmentation eliminate the manual friction of routine contouring and chart checking, the clinical baseline will shift. We will not do the same amount of work with fewer people; rather we will do vastly more sophisticated work with our workforce. Tomorrow's trainees will not be relegated to manual image segmentation or script writing. Instead, they will act as architects and supervisors in roles that demand rigorous knowledge of foundational physics and biology to prevent automation bias and clinical drift. AI is not a replacement workforce but a cognitive multiplier that will catalyze a new era of personalized patient care.

I agree that AI will absorb routine workflows. However, this does not diminish the need for new trainees; rather it elevates the required expertise for entry‐level positions. If automation allows a clinic to routinely implement highly complex, traditionally labor‐intensive techniques like quantitative diagnostic imaging techniques, daily ART, MR‐guided tracking, and FLASH therapy for the broader patient population, the demand for trained experts to commission, validate, and manage these techniques can only grow. This matters at exactly the margin Dr. Xia describes: a department that adopts daily ART or expands SBRT access does not absorb that work with freed‐up time—it commissions new programs, which historically has been the single most reliable driver of new physics hires.

The parallel between corporate tech sector layoffs and clinical healthcare demands needs further comparison. It appears that software giants in the technology sector are reducing employee headcounts after periods of over‐hiring. Healthcare is different; is it even possible to over‐hire in the fight against disease? And if we do reach a point in history where we ultimately cure diseases like cancer in a way that requires fewer healthcare employees, then this should be a welcomed outcome and a pinnacle of human achievement. Until then, there is much more work to do!

## AUTHOR CONTRIBUTIONS

Dongxu Wang proposed the debate topic and performed editing. JX and AM contributed to writing and editing.

## CONFLICT OF INTEREST STATEMENT

Junyi Xia is a co‐founder and CEO of Infondrian, LLC.
